# Sequenced-based paternity analysis to improve breeding and identify self-incompatibility loci in intermediate wheatgrass (*Thinopyrum intermedium*)

**DOI:** 10.1007/s00122-020-03666-1

**Published:** 2020-08-12

**Authors:** Jared Crain, Steve Larson, Kevin Dorn, Traci Hagedorn, Lee DeHaan, Jesse Poland

**Affiliations:** 1 Department of Plant Pathology, 4024 Throckmorton Plant Sciences Center, Kansas State University, Manhattan, KS 66506 USA; 2 USDA-ARS, Forage and Range Research, Utah State University, Logan, UT 84322 USA; 3 AAAS Science and Technology Policy Fellow, USDA-APHIS, 4700 River Road, Riverdale, MD 20737 USA; 4 The Land Institute, 2440 E. Water Well Rd, Salina, KS 67401 USA; 5 Wheat Genetics Resource Center, Kansas State University, Manhattan, KS 66506 USA; 6 Present Address: USDA-ARS, Soil Management and Sugarbeet Research, Fort Collins, CO 80526 USA; 7 Present Address: Quantitative Scientific Solutions LLC, Arlington, VA 22203 USA

## Abstract

**Key Message:**

**Paternity assignment and genome-wide association analyses for fertility were applied to a** ***Thinopyrum intermedium*** **breeding program. A lack of progeny between combinations of parents was associated with loci near self-incompatibility genes.**

**Abstract:**

In outcrossing species such as intermediate wheatgrass (IWG, *Thinopyrum intermedium*), polycrossing is often used to generate novel recombinants through each cycle of selection, but it cannot track pollen-parent pedigrees and it is unknown how self-incompatibility (SI) genes may limit the number of unique crosses obtained. This study investigated the potential of using next-generation sequencing to assign paternity and identify putative SI loci in IWG. Using a reference population of 380 individuals made from controlled crosses of 64 parents, paternity was assigned with 92% agreement using Cervus software. Using this approach, 80% of 4158 progeny (*n* = 3342) from a polycross of 89 parents were assigned paternity. Of the 89 pollen parents, 82 (92%) were represented with 1633 unique full-sib families representing 42% of all potential crosses. The number of progeny per successful pollen parent ranged from 1 to 123, with number of inflorescences per pollen parent significantly correlated to the number of progeny (*r *= 0.54, *p* < 0.001). Shannon’s diversity index, assessing the total number and representation of families, was 7.33 compared to a theoretical maximum of 8.98. To test our hypothesis on the impact of SI genes, a genome-wide association study of the number of progeny observed from the 89 parents identified genetic effects related to non-random mating, including marker loci located near putative SI genes. Paternity testing of polycross progeny can impact future breeding gains by being incorporated in breeding programs to optimize polycross methodology, maintain genetic diversity, and reveal genetic architecture of mating patterns.

**Electronic supplementary material:**

The online version of this article (10.1007/s00122-020-03666-1) contains supplementary material, which is available to authorized users.

## Introduction

Perennial grain crops are posited to provide ecosystem services, such as reduced nitrate leaching and less soil erosion, as well as nutrition for the growing human population (Glover et al. 2010). Yet nearly 80% of the world’s calorie consumption is from annual crops (Pimentel et al. 2012), and the majority of cropping land is planted to annuals (Cox et al. 2010). One of the main challenges in the development of perennial grain crops is their lower yield compared to their annual counterparts (DeHaan et al. 2005; Cox et al. 2006; Kantar et al. 2016). While there has been efforts to develop perennial grain crops for nearly 100 years, there has been little sustained activity (Kantar et al. 2016), with even the most promising candidates only going through 10 or fewer cycles of selection (DeHaan et al. 2018).

One promising species for perennial grain is intermediate wheatgrass (IWG, *Thinopyrum intermedium*) which is an outcrossing perennial grass with a large allohexaploid (2*n* = 6*x* = 42, 12.7 GB (Vogel et al. 1999)) genome similar in size and complexity to bread wheat (*Triticum aestivum*). The inferred origins of the polyploid *T. intermedium* genome have not been entirely consistent but are understood to be an allohexaploid with three distinct subgenomes (Chen et al. 1998; Tang et al. 2000; Mahelka et al. 2011; Wang et al. 2015). It is clear from linkage mapping experiments that IWG shows disomic inheritance patterns similar to allohexaploid wheat (Kantarski et al. 2016). Further clarification of genome relationships between IWG and its diploid relatives is being obtained from the *T. intermedium* Genome Sequencing Project (http://phytozome.jgi.doe.gov/).

With improvement for domestication traits and targeted breeding for yield and agronomics, IWG has strong potential for commercial production. At least three long-term breeding and genomic selection programs have been established, which aim to domesticate and improve IWG as a perennial grain crop marketed as ‘Kernza’ (Cattani 2016; Zhang et al. 2017; Cattani and Asselin 2018; DeHaan et al. 2018). These programs are founded solely or largely on a base population that traces back to 14 individual plants selected at the Rodale Research Center in 1995 (Wagoner 1990; Zhang et al. 2016).

Reflecting limited breeding compared to annual crops, perennial forage crops have less genetic gain compared to annual crops. Casler and Brummer (2008) estimated that perennial crops have achieved 10% of gains made in cereal crops, while Humphreys (1997) estimated that perennial crops have a 4% increase in genetic gain per decade compared to 13.5% gain per decade for annual crops. This smaller increase in gain for perennial forage crops is often attributed to a variety of factors including limited opportunity to exploit heterosis and longer breeding cycle (Humphreys 1997; Casler and Brummer 2008). Although heterosis between inbred lines has been extremely beneficial in many crops, severe inbreeding depression limits development of inbred lines in forage crops (Brummer 1999). Within diploid maize (*Zea mays* L), intensive recurrent selection has removed deleterious alleles while promoting favorable combinations of complimentarily haplotypes across heterotic groups (Walters et al. 1991). However, in autotetraploid alfalfa (*Medicago sativa* L.) the rate of inbreeding depression (30% yield loss in one generation) was far greater than would have been predicted by inbreeding coefficients alone (Busbice and Wilsie 1966), suggesting that purging of deleterious alleles and exploitation of heterosis may be more difficult in polyploids. Additionally, many forage programs introgress new material into their existing breeding program instead of maintaining separate genetic pools which reduce the chances of exploiting maximum heterosis (Brummer 1999).

Self-incompatibility (SI) may be one of the inherent genetic factors limiting forage breeding gains and the development of perennial grain crops. Self-incompatibility limits the ability to develop inbred lines, and it can potentially limit the number of compatible parents in elite breeding populations. Self-incompatibility is well known in the grasses (Poaceae) and is often more prevalent in perennials than annuals (Baumann et al. 2000). The grass family has a gametophytic SI system that is controlled by two independent and multiallelic gene loci (*S* and *Z*) first reported by Lundqvist (1954) and confirmed in perennial ryegrass (*Lolium perenne*) by Cornish et al. (1979). Incompatibility only occurs when the S and Z alleles expressed by the gametophytic haploid pollen genotype have at least one matching S allele and at least one matching Z allele in the diploid pistil parent (Baumann et al. 2000). With a gametophytic SI system controlled by the complimentary action of two genes, there can be differences in fecundity of reciprocal crosses and compatibility based on the pollen donor and recipient (Baumann et al. 2000). This genetic system can also operate in polyploids and previous research has shown that autotetraploids maintain SI (Lundqvist 1957). While much work has been conducted to understand these genes, most studies have relied on populations, typically bi-parental, that have parents with contrasting alleles (Thorogood et al. 2017) with examples including Cornish et al. (1979), Kantarski et al. (2016), Manzanares et al. (2016). The ability to utilize an entire breeding program’s germplasm may provide a better opportunity to identify and study specific SI loci than what is possible with directed crosses which must be compatible (Thorogood et al. 2017).

Research to understand the SI system has revealed that the polyallelic *S* and *Z* loci are located on homoeologous chromosomes 1 and 2, respectively, of diploid *Lolium perenne* (2*n* = 2*x* = 14) (Shinozuka et al. 2010; Manzanares et al. 2016; Thorogood et al. 2017) and rye (*Secale cereale*, 2*n* = 2*x* = 14) (Hackauf and Wehling 2005). Recent studies have shown that it is possible to detect the location and effects of the *S* and *Z* genes in genetically heterogeneous populations by genome-wide association analysis (GWAS), based on the deviations from random mating patterns among parental lines of *Lolium perenne* (Thorogood et al. 2017). The *S* and *Z* genes have been difficult to identify (Thorogood et al. 2017), though it is now believed that a domain-of-unknown-function (DUF) protein, encoded by a *DUF247* gene, acts as the pollen component of the *S* locus on homoeologous chromosome 1 (Manzanares et al. 2016) and that another *DUF247* gene (Shinozuka et al. 2010) or closely linked *ubiquitin*-*specific protease* (*USP*) gene (Hackauf and Wehling 2005) are the best candidate genes for the *Z* locus on homoeologous chromosome 2. However, it is not known how these two orthogenes would operate in allopolyploid species such as IWG even though strong synteny is expected and observed among IWG, barley (*Hordeum vulgare*), *Lolium perenne*, and other grasses (Kantarski et al. 2016; Manzanares et al. 2016; Shinozuka et al. 2010). Moreover, the number and diversity of *S* and *Z* gene alleles in the IWG breeding programs could be a limiting factor in the percentage of observed crosses after a genetic bottleneck of only 14 founder individuals.

One potential avenue to increase genetic gains may be through the utilization of pedigree information in breeding programs. In addition to better understanding the population dynamics, pedigree information has often been used for prediction of genetic value, which is an essential aspect in the improvement of quantitative traits (Crossa et al. 2010). While pedigrees have been invaluable to the breeding and research communities, the ability to maintain pedigrees within breeding populations can be challenging. This is especially true for obligate outcrossing species (Lambeth et al. 2001) which are often bred with random mating in crossing blocks without pollen parent control (Vogel and Pedersen 1993). The progeny observed from random mating is a result of the number of successful pairwise crosses which depend in part on the number and diversity of SI genes, alleles, and genotypes as well as the direction of pairwise crossing for gametophytic SI systems (Thorogood et al. 2017).

Molecular markers can be used to construct pedigrees in outcrossing populations, by allowing paternity to be assigned based on marker genotypes. Paternity analysis has been proposed for a variety of uses within breeding programs including enhancing selections and maintaining pedigrees (Lambeth et al. 2001; Riday 2011; Vleugels et al. 2014; Alam et al. 2018). Within forage and polycross breeding programs, selecting on both parents should theoretically double the rate of genetic gain, compared to selecting on maternal knowledge alone (Fehr 1987; Posselt 2010); consequently, the use of paternity analysis has potential to provide high rates of genetic gain for minimal cost (Li and Brummer 2012). Paternity analysis has been used successfully in several crops including white clover (*Trifolium repens* L.) (George et al. 2018), red clover (*Trifolium pretense* L.) (Riday 2011; Vleugels et al. 2014), Eucalyptus (*Eucalyptus urophylla*) (Grattapaglia et al. 2004), and Timothy grass (*Phleum pretense* L.) (Tanaka et al. 2018). In addition to aiding in breeding, paternity analysis has been used to maintain paternal balance in polycrosses (Tanaka et al. 2018), track pollination events across distances (Isagi et al. 2000; Vleugels et al. 2014), and evaluate relatedness among progeny (Lambeth et al. 2001).

While simple sequence repeat (SSR) markers have been the most common technique for assigning paternity, next-generation sequencing (NGS) can provide thousands of low-cost single nucleotide polymorphic (SNP) markers and SNPs can be used to overcome the main problems of SSR parentage analysis of inadequate marker number and incomplete marker information (Marshall et al. 1998; Jones and Ardren 2003; Pemberton 2008). Typical paternity analysis with SSR markers has used 6–32 markers (Coltman 2005; Dickerson et al. 2005; Riday et al. 2013; Vleugels et al. 2014; Tanaka et al. 2018), but other studies have shown that 60–400 SNP markers could be used to infer parentage in large populations with higher accuracy than SSRs (Anderson and Garza 2006; Thrasher et al. 2018). While there are benefits to paternity analysis, some challenges include cost (Riday 2007) and implementation within the breeding program (Lambeth et al. 2001).

Given these considerations, it would be more feasible to implement paternity testing within a breeding program if the markers were also used for marker-assisted selection or genomic selection (GS). With the ability of NGS to identify and genotype thousands of markers, paternity analysis may become a routine part of outcrossing breeding programs, especially in programs that adopt GS. Thus, the same markers that are being used for selection could also be used to infer parentage and provide pedigree information in breeding programs that use polycross breeding.

Our objectives were to assess how paternity analysis could be incorporated into the IWG GS breeding program. Specifically, we evaluated (1) the ability to determine paternity using GBS markers that often have a large amount of missing data, (2) potential to enhance breeding decisions based on paternity, and (3) whether genetic factors, such as SI gene diversity, are a limiting factor in the number of observed pairwise crosses in the polycrossing stage of this breeding program. Our strategy was to use SNP marker data from implementing GS in an IWG breeding program to construct pedigrees and detect evidence of SI genes that may be limiting the percentage of successful matings in the critical phase of parental polycrossing.

## Materials and methods

### Plant material

Two populations from The Land Institute’s IWG breeding program were used to assess the feasibility of paternity analysis within the breeding program. First, a test population of 380 plants from cycle 5 of the IWG program derived from controlled crosses of 64 cycle-4 parents was used to evaluate the potential of paternity assignment with SNP markers. The parent plants were vernalized and then brought into greenhouses for crossing. Supplemental lighting was provided with 400-W high-pressure sodium lights to provide 16-h day length, and daily watering was provided with flood benches. The temperature in the greenhouse was maintained between 16 and 25 °C. The controlled crosses were made by bagging all inflorescences to prevent cross-pollination. Inflorescences from selected parents for crossing were bagged together using white baguette bags (WebstaurantStore, Lititz, PA) and then agitated daily to ensure pollination (DeHaan et al. 2018). Second, a larger breeding population comprised of 4170 cycle-7 plants derived from an open polycross of 89 cycle-6 parents with uncontrolled pollination was used to evaluate efficacy of random intermating and possibility of limited progeny combinations within the breeding program.

For mating, the cycle-6 parents were first cloned between four and eight times into individual pots. The clones were divided into two groups, with the first group entering the greenhouse 2 weeks before the second group to allow mating of lines with differences in maturity. Then, individual clones of cycle-6 parents were randomly placed on a greenhouse bench and rearranged every three to 5 days during anthesis to enhance the number of random pairwise crosses. Oscillating fans were also used to aid in pollen distribution. The greenhouse climate control system was set to maintain temperatures between 16.7 and 25.0 °C, with an average recorded temperature of 20.6 °C throughout the growing season. Supplemental lighting was provided using 1000-W metal halide lamps set to provide 16-h day length. Lamps were activated whenever the ambient lighting fell below 240 umol m^2^ s^−1^ photosynthetically active radiation (PAR), with lamps providing approximately 160 umol m^2^ s^−1^ PAR. Seed was harvested from each mother plant providing known pedigrees for half-sib families, with unknown pollen parents. Number of inflorescences per clone, seed spike yield in grams, and seed weight in milligrams were recorded from the parental plants in the greenhouse crossing block. The cycle-7 population was initiated with approximately 50 seeds from each maternal genotype, with visual selection removing plants with low seedling vigor, resulting in approximately 45 plants from each mother. These plants were tissue-sampled and genotyped for a total of 4170 cycle-7 plants.

### Genotypic analysis

Genotyping-by-sequencing was applied using a two-enzyme GBS approach similar to the methods of Poland et al. (2012). Sequencing for all individuals was on Illumina HiSeq machines, but the depth of sequencing varied between populations. Cycle-4 and cycle-5 individuals were sequenced at 96 plex, with some plates sequenced twice, giving high coverage for these individuals. Cycle-6 parents were sequenced at 96 plex, while progeny were sequenced at 192 plex. Parents were sequenced at a higher level to obtain less missing data and insure that all parents had sufficient genotyping depth to enhance SNP call accuracy for progeny testing. Single-nucleotide polymorphisms were called using the TASSEL GBSv2 pipeline (Glaubitz et al. 2014) and the version 1.0 draft intermediate wheatgrass genome assembly (access provided by the *T. intermedium* Genome Sequencing Project, https://phytozome-next.jgi.doe.gov/info/Tintermedium_v2_1).

The GBS SNP calling pipeline was combined for both cycle-4 and cycle-5 and cycle-6 and cycle-7 with a total of 131,880 SNPs identified across the populations. Filtering was consistent for each set of data and performed to filter for: (1) tags that aligned to only one location in the reference genome. Using the 64 base pair tag, if a GBS tag aligned to more than one location in the reference genome, it was discarded to prevent potentially combining homeologous loci, (2) a minimum sequencing depth of four tags was required for calling a homozygous genotype. Using a custom Perl script homozygote calls with a tag count (sequence depth), less than four were set to missing. Heterozygous calls were allowed with a minimum of two contrasting tags per locus, (3) less than 70% missing data per SNP, (4) a minor allele frequency (MAF) greater than 0.01, and (5) biallelic SNPs. Multiallelic SNPs and presence/absence variants were discarded. Additionally, individuals that had more than 95% missing data were excluded from further analysis. After filtering, the data sets contained 59,921 SNPs and 444 individuals for cycle-4 and 5. Cycle-6 and 7 contained 27,530 SNPs and 4247 individuals.

### Paternity assignment

Cervus version 3.0.7 (Kalinowski et al. 2007) was used to assign paternity. For all Cervus runs, parameters were set at default values except where noted below. Each Cervus run consisted of completing an allele frequency analysis, followed by a simulation of parentage analysis where the number of potential fathers was set to 64 and 89 (cycle-4 and 5 and cycle-6 and 7, respectively), with a proportion of fathers sampled set to 95%. This parameter allows the software to consider the possibility that the actual father was not genotyped, which was assumed to be possible, but infrequent due to controlled and greenhouse crossing scheme limiting outcrossing outside of the defined crossing block. Proportion of typed loci as the setting for missing data was set to 50% as the GBS markers often contain a large amount of missing data, and this was the maximum amount of missing data possible after filtering (see below). A minimum of 300 typed loci were required for progeny to be analyzed for paternity, and the number of progeny simulated was set to 250,000. The test for self-fertilization option was included in the simulation of paternity to identify any genets that were not cross-pollinated.

Initial runs using the full marker set for cycle-4 and 5 plants resulted in a floating-point overflow error. The working solution was to reduce the marker set. Thus, more stringent filtering was used, resulting in the maximum missing SNPs per loci being 50%. In addition, an index of the percent present of markers and the average read depth of each SNP marker was used to identify highly reliable markers in the population. The minimum MAF for progeny testing was chosen above reported literature values for paternity testing of 0.02 (Gill et al. 2003).

The cycle-4 and 5 and cycle-6 and 7 had different sequencing coverage, resulting in 32,241 markers that passed all filters for cycle-4 and 5 compared to cycle-6 and 7 which only had 10,284 markers pass all previous filters. To maintain a small set of marker numbers, the final marker set consisted of 2500 markers from cycle-6 and 7 with the highest index values. The final markers from cycle-4 and 5 markers were chosen at random from the SNPs passing all filters and having an index value equal to the minimum value of cycle-6 and 7. Within cycle-4 and 5, we also masked the mothers and assigned maternity based on the markers.

The *vegan* R package (Oksanen et al. 2017) was used to determine the Shannon’s diversity index (*H*) to compare diversity of the random polycross to direct crossing in terms of each program’s ability to obtain a diverse set of progeny for selection.

### Identification of putative S and Z SI genes

We utilized identified candidate genes for the SI loci from previous studies and positioned these gene sequences on the reference IWG assembly. *S* and *Z* candidate genes included the *Brachypodium* Bradi2g35750.2 and Bradi5g23930.2 gene models corresponding to the putative *S*-*DUF247* and *Z*-*DUF247* loci on *Lolium perenne* linkage group 1 and 2, respectively (Thorogood et al. 2017); the *Lolium perenne S*-*DUF247* candidate gene (Manzanares et al. 2016); the rice (*Oryza sativa*) *Os04g0647701 DUF247* gene corresponding to the *Lolium perenne Z*-*DUF247* candidate gene (Shinozuka et al. 2010); and a *ubiquitin*-*specific protease* (*UBP*) gene (*OSJNBa0070O11.10*) on rice BAC OSJNBa0070O11 corresponding to the TC116908 sequence-tagged site PCR marker for the rye *Z*-*UBP* candidate gene on chromosome 2RL (Hackauf and Wehling 2005).

The putative *S* and *Z* DNA and protein sequences were aligned to the annotated version (2.1) of the draft genome assembly of intermediate wheatgrass using BLASTN or BLASTP (Altschul et al. 1990), respectively, with an E-value threshold of 1E − 60. Linkage groups 1 and 2 of *Lolium perenne* correspond to allohexaploid IWG chromosomes 1–3, and 4–6, respectively (Kantarski et al. 2016).

### Genome-wide association analysis of progeny

We used two different approaches to determine if genetic factors were preventing certain parental combinations within the polycross, and thus limiting the application of random mating within the breeding program. Both approaches leveraged information about the polycross and the cycle-7 assigned paternity. Since all cycle-7 plants were the progeny from 89 cycle-6 parents, a progeny matrix representing all 89 × 89 = 7921 possible crosses (full diallel) could be made to record the number of progeny obtained from each cross. The progeny matrix was encoded with the number of progeny observed from the paternity assignment, or if no progeny was observed, a zero was recorded. This progeny matrix formed the basis of the phenotypic response to investigate evidence for non-random mating.

The first approach to study the observed mating patterns relied on making in silico genotypes of all potential progeny. Using genotypes from GBS SNP markers of the 89 parents, each *F*_1_ genotype of possible progeny (observed and unobserved) could be estimated. Before making in silico progeny genotypes, missing SNP markers were imputed using Beagle version 4.1 (Browning and Browning 2016). For each of the 9358 loci, the progeny loci were encoded based on the hypothesized action of a single gametophytic SI reaction (Newbigin et al. 1993). A dominant coding system resulted in two possible genotype classifications for each marker, (1) where the female parent contained the exact alleles as the pollen parent alleles, which was coded as − 1; (2) where female and pollen parent alleles were contrasting, coded as + 1, Table 1. Using a priori information about the SI system, we postulated that if SI occurred at a marker locus, individuals coded as − 1 would be incompatible and lack observed phenotypes (progeny from particular parental combination), whereas individuals with the + 1 would be compatible and result in observed individuals from this progeny cross. As IWG has a two loci (*S* and *Z*) SI system (Baumann et al. 2000), this coding system allows parents to have a homozygous marker state, but implicitly suggest that this marker state would not be favorable for progeny. All markers were encoded with this system, allowing us to test individual markers for distortion, yet retaining information about the entire genome (*S* and *Z loci*) which would affect if progeny was observed. For example, we assumed that in the presence of SI and no observed progeny, the two loci would be the same (incompatible), while for observed progeny, the loci between parents would differ. A GWAS was performed using the dominant marker coding matrix for all 7921 in silico progeny with the phenotypes coded as 0 if no progeny from a particular cross-combination was observed, and 1 if progeny from a cross was observed.

**Table 1:** Gametophytic compatibility outcomes corresponding to three possible parental genotypes for a biallelic, self-incompatibility (SI) single-loci

Male parent genotype	Female parent genotype	Progeny loci genotype code	Expected SI phenotype
SI_1_SI_1_ = − 1	SI_1_SI_1_ = − 1	− 1	I
SI_1_SI_1_ = − 1	SI_1_SI_2_ = 0	− 1	I
SI_1_SI_1_ = − 1	SI_2_SI_2_ = 1	+ 1	C
SI_1_SI_2_ = 0	SI_1_SI_1_ = − 1	+ 1	C
SI_1_SI_2_ = 0	SI_1_SI_2_ = 0	− 1	I
SI_1_SI_2_ = 0	SI_2_SI_2_ = 1	+ 1	C
SI_2_SI_2_ = 1	SI_1_SI_1_ = − 1	+ 1	C
SI_2_SI_2_ = 1	SI_1_SI_2_ = 0	− 1	I
SI_2_SI_2_ = 1	SI_2_SI_2_ = 1	− 1	I

Progeny genotypes with − 1 are assumed incompatible (I) as female mother contains the exact alleles as the pollen parent, and 1 is compatible (C) where the female mother contains contrasting alleles to the pollen parent. Coding system was used for each of 9358 biallelic single-nucleotide polymorphic markers to develop full in silico genomic profiles of progeny, with the expected phenotype corresponding to I or C with SI occurring

We also evaluated a method used by Thorogood et al. (2017) where principal component analysis (PCA) was performed on the pollination matrix and then principal components (PCs) were used as the phenotypic response to identify SI locations. The pollination matrix was observed semi-in vivo pollen tube germination, with scoring providing evidence of the compatibility of each mating. We used the progeny matrix analogously to the pollination matrix of Thorogood et al. (2017) where observed progeny was evidence of compatibility and unobserved progeny was assumed incompatible. Using the 89x89 progeny matrix where the value of the matrix cell was the observed progeny from each parent pair combination, the first 3 PCs (PC1, PC2, PC3) were evaluated using the prcomp function in R (R Core Team 2017). These component scores were then modeled in a GWAS with the recorded genotypes of the 89 parents, with each PC (1 through 3) corresponding to models B, C, and D, respectively.

The *rrBLUP* package (Endelman 2011) was used for the GWAS analysis. A total of 9358 imputed markers that were assigned to chromosomes from the IWG genome sequence were used for all GWAS analysis. Across the genome, the median SNP density was three per megabase (Fig. S1). The statistical model to evaluate the GWAS was based on the mixed-linear model from Yu et al. (2006), Eq. (1), that can account for both population structure and kinship.

1 $$y = X\beta + Zg + S\tau + \varepsilon$$

In Eq. (1), *y* is an *n* × 1 vector of phenotypic response, $X\beta$ are fixed factors modeling population structure using PCA, where *X* is an *n* × *f* matrix, where n is the number of individual genets and *f* is the number of fixed effects and $\beta$ is an *f* × 1 vector of fixed effects, *Zg* accounts for the random effect of each line with *Z* being the genomic relationship matrix of size *n* × *n* and *g* is an *n* × 1 vector of polygenic background effects, $S\tau$ is the fixed effect response for each marker tested independently, where *S* is an *n* × 1 vector of marker scores and $\tau$ is marker effect treated as a scalar, and $\varepsilon$ is an *n* × 1 vector of random error. Population structure was accounted for by using 5 PCs. The model compression was set at the optimum level using ‘population parameters previously determined’ (P3D) (Zhang et al. 2010). To control for multiple testing on a genome-wide basis, the false discovery rate (FDR) (Storey and Tibshirani 2003) was set to 0.05, and QQ plots were evaluated to assess model fit (Fig. S2). Power analysis was conducted following the methods of Wang and Xu (2019). On chromosomes harboring a putative SI gene from BLASTN or BLASTP hits (chromosomes 1, 2, 3, and 6), a chromosome-wide FDR of 0.05 was also established. Plots were made using the *qqman* (Turner 2017) and the *CMplot* (Yin 2019) R packages. All data analyses were completed in R (R Core Team 2017).

#### Data availability

All genotypic data have been placed in the NCBI sequence read archive (SRA) (https://www.ncbi.nlm.nih.gov/bioproject/) as BioProject accession number PRJNA563706. Phenotypic data and scripts to complete all analysis have been placed in the Dryad digital repository 10.5061/dryad.0cfxpnvz3.

## Results

### Paternity analysis for known populations

To evaluate the potential to use GBS SNP markers, which often have large amounts of missing data, we assigned paternity using markers for plants from cycles 4 and 5 with known pedigrees. This group of individuals consisted of 64 parents and progeny in full-sib families from controlled pairwise crosses. Using Cervus, we masked the recorded male parent and produced matching results from 92% of the progeny based on molecular markers alone. This assignment was made using 2500 markers with an average read depth of 13.3 reads per marker per individual. Of the 380 progeny, 350 were assigned parentage in agreement with breeding records. Of the 30 mismatches, Cervus assigned 15 to self-pollinations (< 4%), which is possible and indistinguishable from recorded pedigree records. The remaining true errors totaled approximately 4% (15 of 380) and included a parent that had no relationship with the recorded parent in 13 instances, and two of the mismatches were assigned to pollen donors that had a full-sibling or closer relationship according to the recorded parent pedigrees.

Additionally, masking the mother instead of the male parent resulted in a slightly higher assignment rate of 94% compared to 92% paternity assignment. Within cycle-6 and 7, there were 124 individuals from direct crosses representing three half-sib families with an assignment rate of 74% agreement to the pedigree (92 males in agreement, data not shown). Of the mismatches 15 were assigned to potential males that were half-sibling or closer relatedness to the recorded father.

### Paternity analysis in *polycross* population

**Fig. 1:**
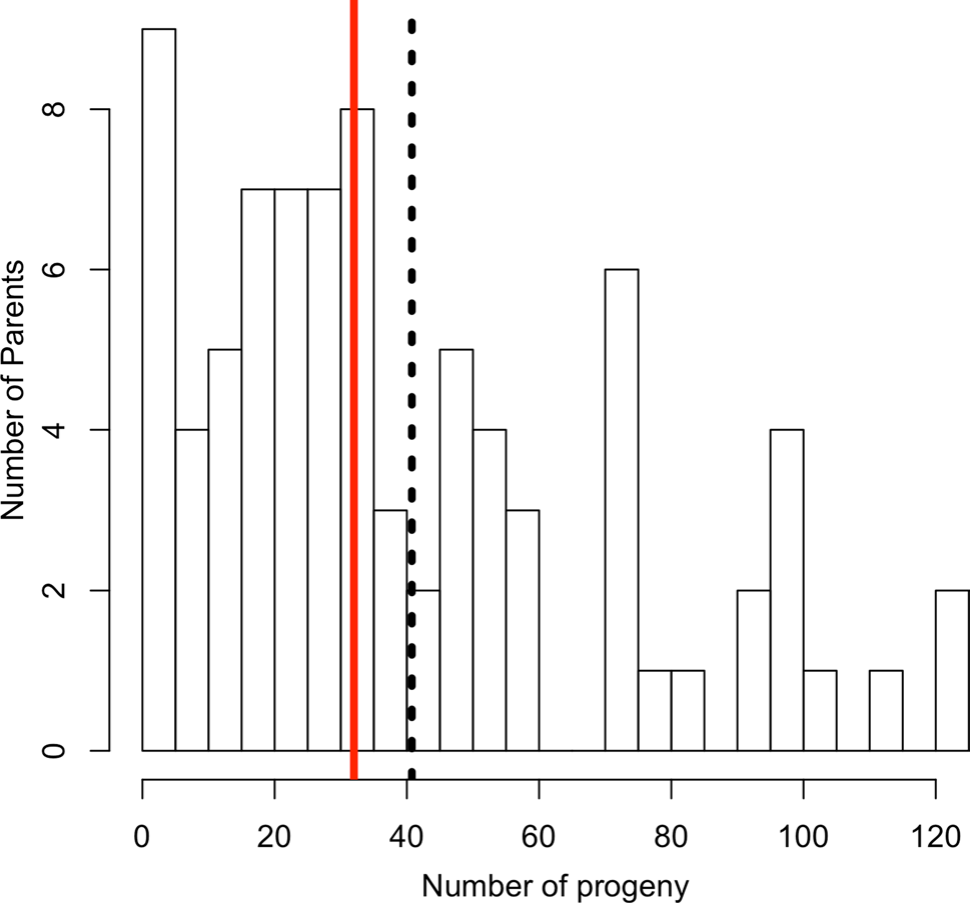
Histogram showing the distribution of the number of progeny of each pollen parent in a polycross breeding program, where paternity was determined through SNP markers. Red vertical line is median value (32), and the dashed black line is the average (41) (color figure online)

**Fig. 2:**
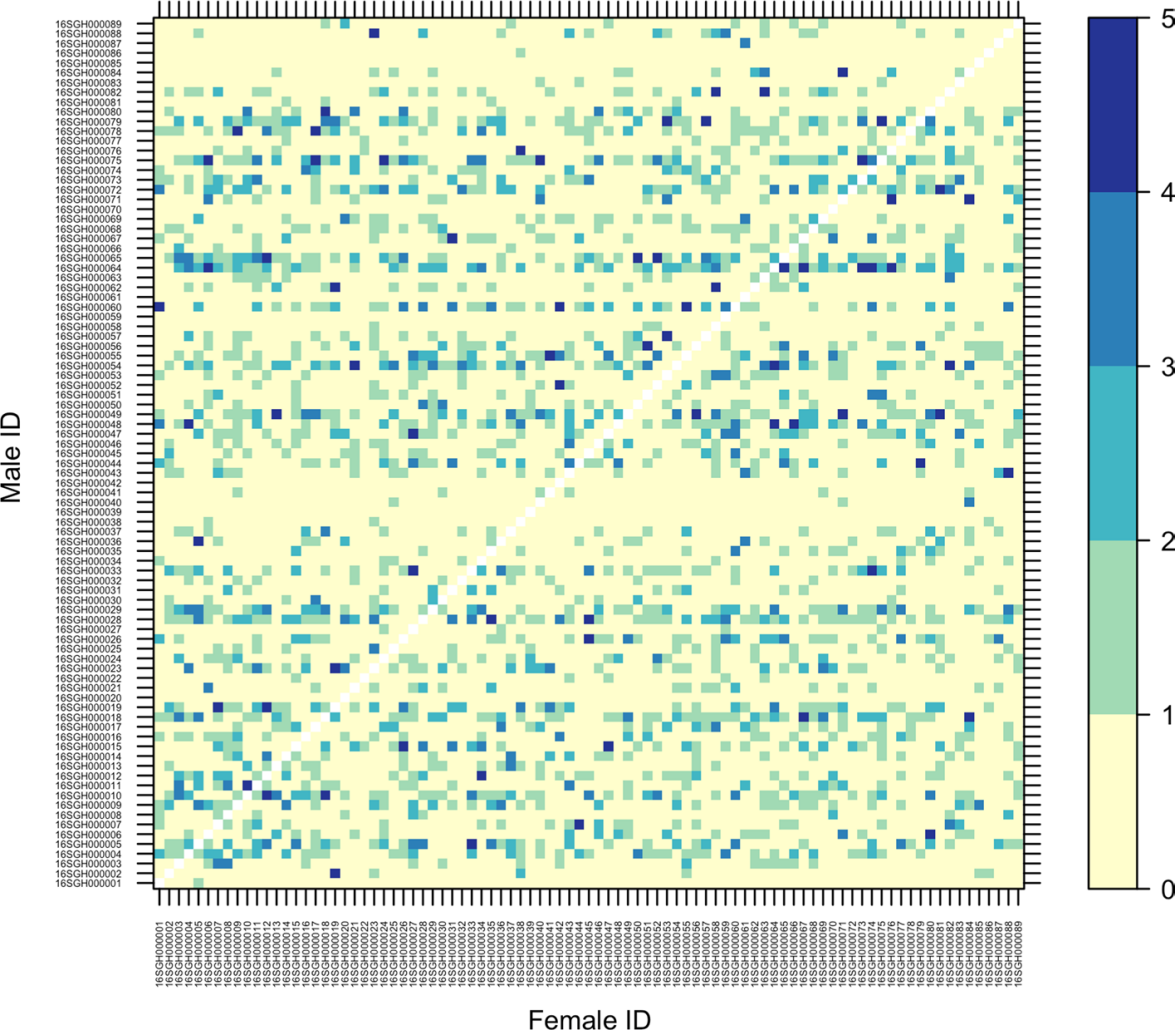
Matrix showing the number of progeny per cross with increasing color gradient, white diagonal is unobserved self-progeny; unobserved combinations are in tan, while crosses with five or more progeny are in dark blue. Female plants are on the *x*-axis with approximately equal distribution per line, observed vertically resulting from the population sampling. Male plants are in the *y*-axis and read horizontally. Some males such as 116SGH000028 crossed with many females, while other such as 16SGH000086 did not sire progeny (color figure online)

Of the 4158 individuals that passed filters for genotyping, we assigned paternity to 3342. There were no self-fertilizations observed among this group of individuals, and the average read depth of markers used for analysis was 6.5 reads per marker per individual. Of the 89 potential fathers, 82 parents sired progeny, with the number of offspring for successful pollen parent ranging from 1 to 123, with a median of 32 progeny per father (Fig. 1). We observed 1949 (25%) of the 7832 total potential combinations considering reciprocal crosses unique (Fig. 2), and 1633 (42%) of the 3916 parental pair combinations with a median of one individual per family and a range of 1–50 individuals per family. For the polycross of 89 parents, *H* was 7.33 considering unique combinations.

### Putative S and Z SI gene identification

The *S*-*DUF247* candidate gene query sequences aligned to a total of three loci on chromosomes 1, 2, and 3 of homoeologous group 1 with *E*-values and alignment scores that were substantially higher than any other BLASTN hits (Table 2). Active transcripts and structurally annotated gene models Thint.01G0027300 and Thint.03G0317600 were found on chromosomes 1 and 3, respectively, with an in active transcript model Thintv21023223m on LG2. Likewise, the *Lolium perenne* LpSDUF247 peptide sequence aligned to predicted peptide sequences of the Thint.01G0027300 and Thint.03G0317600 loci on chromosomes 1 and 3, respectively, with considerably better *E*-values and alignment scores than any other BLASTP hits. The inactive Thintv21023223m locus on chromosome 2 did not have a predicted protein sequence; therefore, it was not detectable in the BLASTP search using the *Lolium perenne* LpSDUF247 peptide sequence. Putative IWG *S*-*DUF247* genes, Thint.01G0027300 and Thint.03G0317600 on chromosomes 1 and 3 of homoeologous group 1, have functional annotation that is orthologous to the homoeologous chromosome-1 *Lolium S*-*DUF247* gene (Table 2).

**Table 2:** Putative S and Z SI gene alignment to the intermediate wheatgrass (IWG) genome and genome-wide association analysis (GWAS) markers with false discovery rate (FDR) values less than 0.05

IWG_locus	Chr	Position	Homologous Group	− Log(p)	GWAS Model	BLAST type	BLAST_Database (IWG V2)	BLAST hit (IWG V2 gene)	Query sequence	Query citation
S01_12234882	1	12234882	1	6.11	A					
**S-DUF247**	**1**	**18026290**	**1**			**DNA**	**Chr01:18026290..18027884**	**Thint.01G0027300**	**Bradi2g35750.2**	**(Thorogood et al.** 2017**)**
**S-DUF247**	**1**	**18026290**	**1**			**Protein**	**Chr01:18026290..18027884**	**Thint.01G0027300**	**LpSDUF247**	**(Manzanares et al.** 2016**)**
S01_42697298	1	42697298	1	5.55	B					
S01_339101017	1	339101017	1	3.87^a^	A					
**Z-UBP**	**1**	**397475588**	**1**			**DNA**	**Chr01:397475588..397478921**	**Thint.01G0459000**	**OSJNBa0070O11.10**	**(Hackauf and Wehling** 2005**)**
**Z-DUF247**	**1**	**397625114**	**1**			**Protein**	**Chr01:397625114..397629144**	**Thint.01G0459200**	**Os04g0647701**	**(Shinozuka et al.** 2010**)**
**Z-DUF247**	**1**	**397627428**	**1**			**DNA**	**Chr01:397627428..397627858**	**Thint.01G0459200**	**Bradi5g23930.2**	**(Thorogood et al.** 2017**)**
**Z-UBP**	**2**	**110208369**	**1**			**DNA**	**Chr02:110208369..110210461**	**Thiinv2.1_pg948986.valid.m1**	**OSJNBa0070O11.10**	**(Hackauf and Wehling** 2005**)**
**Z-DUF247**	**2**	**110318091**	**1**			**Protein**	**Chr02:110318091..110320811**	**Thint.02G0187000**	**Os04g0647701**	**(Shinozuka et al.** 2010**)**
**Z-DUF247**	**2**	**110337256**	**1**			**Protein**	**Chr02:110337256..110339970**	**Thint.02G0187300**	**Os04g0647701**	**(Shinozuka et al.** 2010**)**
**Z-DUF247**	**2**	**110338032**	**1**			**DNA**	**Chr02:110338032..110337540**	**Thint.02G0187300**	**Bradi5g23930.2**	**(Thorogood et al.** 2017**)**
S02_124049901	2	124049901	1	4.32^a^	A					
**S-DUF247**	**2**	**148853965**	**1**			**DNA**	**Chr02:148853965..148855542**	**Thintv21023223m**	**Bradi2g35750.2**	**(Thorogood et al.** 2017**)**
S02_333426395	2	333426395	1	4.46^a^	D					
S02_357481632	2	357481632	1	4.40^a^	A					
S02_408872524	2	408872524	1	4.10^a^	D					
S03_77463845	3	77463845	1	3.94^a^	A					
S03_161965824	3	161965824	1	3.62^a^	A					
**S-DUF247**	**3**	**323947299**	**1**			**DNA**	**Chr03:323947299..323949086**	**Thint.03G0317600**	**Bradi2g35750.2**	**(Thorogood et al.** 2017**)**
**S-DUF247**	**3**	**323947299**	**1**			**Protein**	**Chr03:323947299..323949086**	**Thint.03G0317600**	**LpSDUF247**	**(Manzanares et al.** 2016**)**
S04_134219385	4	134219385	2	4.40	B					
S05_184724149	5	184724149	2	5.29	A					
S06_50454994	6	50454994	2	4.20^a^	A					
S06_234033536	6	234033536	2	4.63^a^	D					
S06_409578239	6	409578239	2	4.10^a^	B					
S06_486347536	6	486347536	2	4.06^a^	B					
S06_495791921	6	495791921	2	6.90	B					
S06_495791961	6	495791961	2	6.90	B					
S06_540749817	6	540749817	2	3.29^a^	B					
**Z-DUF247**	**6**	**560275047**	**2**			**Protein**	**Chr06:560275047..560275943**	**Thint.06G0672200**	**Os04g0647701**	**(Shinozuka et al.** 2010**)**
**Z-DUF247**	**6**	**560953363**	**2**			**Protein**	**Chr06:560953363..560954970**	**Thint.06G0672700**	**Os04g0647701**	**(Shinozuka et al.** 2010**)**
**Z-UBP**	**6**	**560963674**	**2**			**DNA**	**Chr06:560963674..560966813**	**Thint.06G0672800**	**OSJNBa0070O11.10**	**(Hackauf and Wehling** 2005**)**
S10_488147266	10	488147266	4	4.77	B					
S11_209271814	11	209271814	4	6.88	B					
S12_13283789	12	13283789	4	5.58	C					
S18_38158835	18	38158835	6	5.43	B					
S20_276537900	20	276537900	7	5.64	C					
S20_461811575	20	461811575	7	5.11	B					

GWAS models: A. GWAS using in silico progeny genotype encoding for a single-loci, self-incompatibility gametophytic system for observed progeny combinations, B. principal component analysis (PCA) of progeny matrix principal component (PC) 1, C. PCA of progeny matrix PC 2, D. PCA of progeny matrix PC 3

Bold items are putative BLASTN and BLASTP hits

^a^Significant for chromosome FDR = 0.05

The *Z*-*DUF247* and *Z*-*UBP* candidate genes, corresponding to the *Lolium* chromosome-2 Z locus, aligned to closely linked loci (less than 1.1 Mb apart) on each of three IWG chromosomes including chromosomes 1 and 2 of homoeologous group 1 and chromosome 6 of homoeologous group 2 (Table 3). The rice *OSJNBa0070O11.10* gene corresponding the rye *Z*-*UBP* locus had a total of three alignments to chromosomes 1, 2, and 6 (Table 2) with *E*-value and alignment scores than any other BLASTN hits. However, only chromosomes 1 and 6 had annotated *UBP* genes that appeared functional (Table 2). The rice Os04g0647701 protein and *Brachypodium Bradi5g23930.2* gene sequences corresponding to the *Lolium perenne Z*-*DUF247* candidate gene aligned to one annotated *DUF* gene on IWG chromosome 1 and two annotated *DUF* genes on IWG chromosomes 2 and 6 (Table 2).

**Table 3:** Advantages and disadvantages of direct cross-breeding and polycross by random intermating for the breeding programs

	Direct Crosses	Random intermating
Advantages	Large family size Known pedigree Make targeted/desired cross	Large number of families Resource efficient Infer pedigree from markers
Disadvantages	Resource-intensive Limited by available resources	May not observe desired combination Must use molecular markers Small family size

### GWAS of progeny

**Fig. 3:**
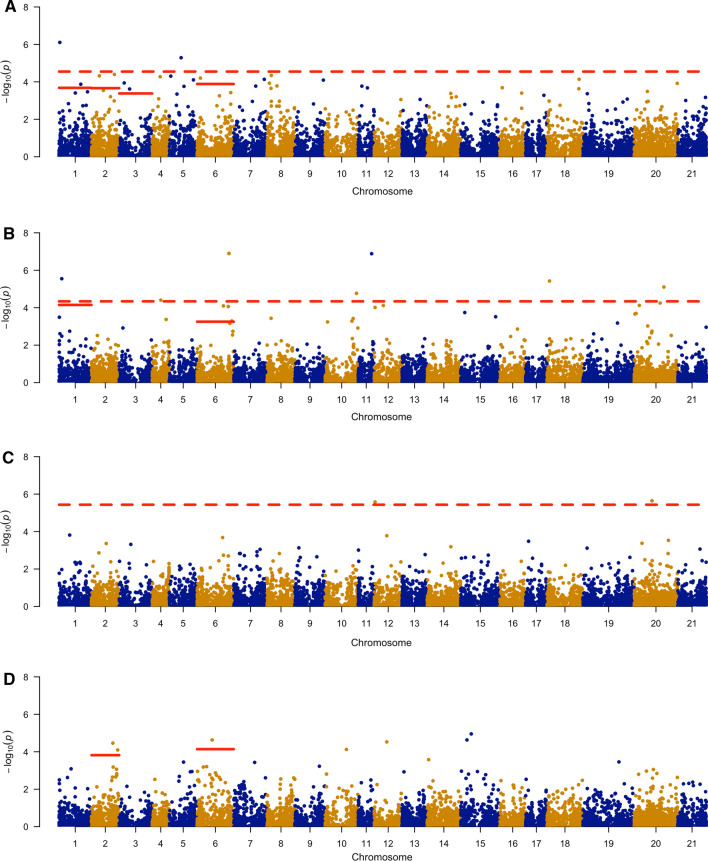
Manhattan plot of genome-wide association analysis (GWAS) of four different models with 9358 markers. The dashed red horizontal line represents the false discovery rate (FDR) of 0.05 controlling for multiple comparisons across the entire genome. Solid red lines represent the FDR of 0.05 for multiple comparisons for each chromosome and were only considered for chromosomes harboring a putative SI gene (chromosomes 1, 2, 3, and 6). Panels represent: **A** GWAS using in silico progeny genotype encoding for a single-loci, self-incompatibility gametophytic system for observed progeny combinations, *n* = 7921. **B** Principal component analysis (PCA) of progeny matrix principal component (PC) 1. **C** PCA of PC 2. **D** PCA of PC 3, *n* = 89 (color figure online)

A progeny presence/absence coding system and three PCs of the progeny matrix were used to conduct a GWAS analysis for non-random mating by association of presence or absence of progeny between a given parent combination and the imputed markers scores of that respective hybrid combination. There were a total of 12 markers with significant mating effects (FDR < 0.05) controlling for multiple comparisons across the entire genome, including six markers on homoeologous groups 1 or 2 of *Lolium perenne* (Table 2; Fig. 3). In addition, 12 other markers had significant mating effects (FDR < 0.05) across chromosomes with putative SI genes (chromosomes 1, 2, 3, and 6).

Several markers were associated with the mapped loci of the putative *S* and *Z* SI genes. On chromosome 1, the Thint.01G0027300 *S*-*DUF247* gene was flanked by the GWAS identified markers S01_12234882 and S01_42697298 at 5.7 MB and 26.6 MB, respectively. The marker S01_339101017 was 58.4 MB from the Thint.01G0459200 *Z*-*DUF247* and Thint.01G0459000 *Z*-*UBP* genes on chromosome 1, Table 2. The marker S02_124049901on chromosome 2 was in between the Thint.02G0187300 *Z*-*DUF247* gene and the Thintv21023223m *S*-*DUF247* gene at distances of 13.7 MB and 24.8 MB, respectively. On chromosome 6, the *Z*-*DUF247* Thint.06G0672200 gene was 19.5 MB from GWAS marker S06_540749817. Other markers associated with this gene were S06_495791921 and S06_495791961, 64.5 MB and S06_486347536 at 73.9 MB in distance.

Other markers were associated with progeny combinations including a marker on chromosome 3, the S03_161965824. Two markers passing the FDR threshold (0.05) were located on linkage groups 4 and 5 although there were no SI gene alignments on this homoeologous group-2 chromosome. Aside from markers located on homeologous group 1 and 2 chromosomes (IWG chromosomes 1-6), six significant markers were located on other chromosomes representing homeologous groups 4, 6, and 7 (IWG chromosomes 10, 11, 12, 18, 20).

## Discussion

### SNP markers for paternity analysis

The accuracy of SNP-based paternity assignments varied from 74 to 94% in progeny from controlled crosses with a recorded pedigree. In some unsuccessful cases, the inferred parent was a full-sibling or highly related to the recorded parent. Instances where a relative was assigned paternity instead of the known father could be due to the similarity that relatives would share, with a potential solution to increase marker density through higher sequencing coverage or filtering on more informative markers to determine the most likely parent. However, in most cases the incorrect assignment showed little in common with the recorded parent. These could likely be the result of pollen contamination or seed mixtures, though spurious results from the genotyping and parentage assignment are also possible.

Sequence coverage may also explain the higher rate of assignment in cycles 4 and 5 compared to cycles 6 and 7, as the former had on average double the read depth leading to more and higher quality SNP calls. With our sequence coverage being higher in parents than progeny, if any progeny was selected for use in the breeding program that did not have assigned paternity, it could be resequenced at greater coverage to assign paternity. Additionally, a more informative subset of markers based on sequence depth for that parent could be chosen, allowing paternity to be assigned.

While we have tested Cervus in this present study, there also exist a number of other paternity software programs. Recent advancements by Whalen et al. (2019) have resulted in AlphaAssign, a python program for paternity assignment with SNP data. Other programs include Sequoia (Huisman 2017) and paternity assignment that makes use of the genomic relationship matrix (Grashei et al. 2018). Any of these programs may provide sufficient results, and breeding programs could use the package that is user friendly and meets their specific needs. If one program is providing insufficient data, other programs may be evaluated to increase paternity assignment or resolve discrepancies.

Mismatches where an unrelated male was assigned paternity are most likely the result of pedigree errors due to cross-fertilization from pollen contamination in the controlled crosses or mislabeling of plants. Of the 15 mismatches, two families (seven plants) had the same candidate males assigned, providing evidence of a mislabeled sample. One other recorded family (five plants) had two unique parents, suggesting possible pollen contamination. Within the breeding program, many sampling stages could result in errors such as crossing, harvesting seed, threshing, cleaning, data entry, and planting. Additionally, as IWG is outcrossing, it is possible that random pollen caused fertilization rather than the intended parents, especially if the intended cross involved incompatible SI genotypes, resulting in unknown parentage reflected by pedigree errors. While the exact nature of the misassignments may not be known, the inferred parentage rate is sufficiently high to be used within breeding programs and may actually be more accurate than pedigrees from controlled crosses given the many possible places for errors in crossing and record keeping.

### Paternity analysis within breeding programs

The results from the known populations show that SNP markers can be successfully used for paternity assignment. Within the IWG breeding program, this allows crossing to be performed randomly without the time and labor associated with pairwise controlled crosses. In addition to cost savings, the number of families observed (1633 different families) was higher than the number of controlled crosses that our program could effectively manage (700 families per year). While achieving a higher number of potential crosses is advantageous, it is also important that the representation among these crosses is balanced. To ensure that the polycross was performing as well or better than direct crossing in this regard, Shannon’s diversity index was calculated for each crossing method. At a maximum, using 89 parents and assuming reciprocal crosses are unique, if all crosses could be made and equal progeny obtained *H *= 8.98. Using direct crossing and allotted resources to make 700 crosses, would allow all progeny to be represented equally and achieve an index of *H* = 6.54. Using the polycross method resulted in 1949 unique crosses and *H* = 7.33, providing evidence that the polycross not only outperformed direct crossing in terms of unique combinations but also in terms of balanced representation of the starting parental lines. Direct crossing would require over 1500 crosses to obtain the same diversity value, more than a doubling of available resources. This higher rate of genetic diversity provides more unique combinations, allowing for desirable plants to be identified and increasing the rate of genetic gain. The use of molecular markers also allows pedigrees to be maintained with uncontrolled pollinations and can even be used to identify unknown or mislabeled plants.

While there are many advantages of random intermating, breeders should be aware of potential issues, especially that not all crosses may be compatible in species such as IWG. To use paternity analysis successfully, germplasm must be genotyped, but in programs utilizing GS the molecular data may already exist. For desired crosses, random mating increases the risk that target crosses may not occur by random chance, and finally, the family size of any particular cross may only be one or a few plants. In such a situation, the breeder could endeavor to make a few controlled pairwise crosses to obtain the most desirable crosses, while allowing the rest of the parents to randomly intermate. If a breeder desires a large family for evaluation or QTL mapping, direct crosses would still be preferable. For the IWG breeding program, advantages and disadvantages of using molecular markers for paternity analysis are summarized in Table 3.

For a breeding program that is already using molecular markers, paternity analysis may be able to provide new insights into optimizing the program. Based on the number of progeny observed per father (Fig. 1), there were clearly differences in fecundity. To obtain the most diverse set of germplasm, it is desirable that each male parent is represented in the progeny to a similar degree. Similar representation of progeny will prevent the loss of genetic diversity by maintaining the effective population size (Falconer and Mackay 1996). From data collected on the parents, the natural log of offspring and the number of inflorescences resulted in correlation of *r *=0.54 (*p* < 0.001, Fig. S3). For future cycles, maintaining similar numbers of inflorescence per parent may help achieve a more representative balance between parents and their offspring.

### Putative S and Z SI gene location

Identification of two putative *S*-*DUF247* genes, Thint.01G0027300 and Thint.03G0317600 located on chromosomes 1 and 3 of IWG homoeologous group 1, showed functional annotation and map positions that seem to be orthologous with the *S*-*DUF247* candidate gene (Manzanares et al. 2016) on *Lolium* chromosome 1. The close linkage of *Z*-*DUF247* and *Z*-*UBP* genes on IWG chromosomes 1, 2, and 6 is consistent across diverse taxa (Hackauf and Wehling 2005; Shinozuka et al. 2010; Manzanares et al. 2016; Thorogood et al. 2017). The locations of the *Z*-*DUF247* and *Z*-*UBP* genes on chromosome 6 of IWG homoeologous group 2 were presumably orthologous to the corresponding loci on *Lolium* homoeologous chromosome 2 (Hackauf and Wehling 2005; Shinozuka et al. 2010; Thorogood et al. 2017), but the locations of these genes on chromosomes 1 and 2 of IWG homoeologous group 1 were surprising. Previous studies showed synteny and colinearity of numerous genes in the *S* and *Z* regions across diverse taxa including *Brachypodium*, *Lolium prenne*, rice, rye, and sorghum (*Sorghum bicolor*) (Hackauf and Wehling 2005; Shinozuka et al. 2010; Thorogood et al. 2017). Thus, the location of putative *Z*-*DUF247* and *Z*-*UBP* genes on IWG chromosomes 1 and 2, in the draft genome sequence of IWG, may warrant scrutiny, but it is also quite possible that the activity and location of these genes has been disrupted during the allopolyploid evolution of IWG. Putative SI genes were located in the IWG genome, yet given the allohexaploid nature of IWG, it is not known how these ortho- or paralogs would operate. It is possible that the duplicate genes would degenerate reverting to only two functional loci and a genetic system comparable to the diploids or that SI activity might be retained at multiple loci across the subgenomes (Veitia 2005).

### Association mapping of compatibility

We explored two separate approaches to perform GWAS for self-incompatibility using the number of observed progeny. Even though our observed progeny only consisted of 4170 random plants, the GWAS results from both methods consistently show that markers located near putative SI genes are associated with the observation (or lack thereof) of progeny combinations. While both methods, GWAS on in silico progeny genotypes and GWAS on the PCA of the progeny matrix, identified markers near SI genes, each method led to identification of different markers. For example, the *S*-*DUF247* on chromosome 1 was flanked by the in silico GWAS marker on one side and the marker from the GWAS of the PCA progeny on the other, with no common markers identified between the two methods. This could be expected based on the amount of missing or inferred data and the methods. Power analysis revealed that while GWAS on the in silico progeny was > 85%, the power of the GWAS PCA progeny was below this threshold. Additionally, we did not find extensive linkage disequilibrium (Fig. S4). Given the low levels of linkage disequilibrium and limited power of the GWAS on the PCA progeny, it is not surprising that different markers were identified by each method.

The close association of the most prominent GWAS marker near the *S*-*DUF247* gene, Thint.01G0027300, on IWG chromosome 1, provides compelling evidence that we are correctly associating genetic effects with the presence of viable progeny and that this is an active SI locus, like the *Lolium S*-*DUF247* gene. Other GWAS markers were also located near putative *S* and *Z* SI genes, suggesting that the diversity of *S*-*DUF247, Z*-*DUF247,* and *Z*-*UBP* alleles may be a limiting factor in cross-compatibility of genotypes in the IWG breeding program.

Although we focused on markers located near putative SI genes, there were several markers that were not associated with known SI genes or homeologous groups with reported SI activity. One possible explanation is that the draft genome still contains many potential errors and misplaced scaffolds. As the genome is further refined, the position of significant markers may change. Another potential explanation is that these markers are associated with traits such as maturity, pollen production, plant height, tiller number which impact the probability of obtaining progeny or postzygotic incompatibility. While the cycle-6 parents were staged in two groups to overcome differences in maturity, there still may be effects of flowering time associated with the observed progeny combinations. In maize (*Zea mays*), flowering time has been shown to lead to assortative mating (Gutierrez and Sprague 1959; Ennos and Dodson 1987). Endosperm incompatibility has been shown to reduce viable seed in many interspecific crosses of differing ploidy levels (Martínez-Reyna and Vogel 2002; Lafon-Placette and Köhler 2016). While the crosses in this experiment should not vary in ploidy level, postzygotic barriers have been observed in crosses of *Arabidopsis thaliana* (Wolff et al. 2015), and it is possible that some of the significant marker trait associations for fecundity are effected by these or similar genetic factors. In fact, the number of inflorescences per parent was positively correlated with number of progeny, suggesting pollen production may be one of the factors associated with non-random mating in this study. Traits such as plant height, pollen production, maturity, and propensity to tiller may need to be considered in an effort to obtain the maximum number of progeny combinations from polycrosses in IWG. While these uncontrolled factors may lead to bias in observed matings, the significance of the GWAS hits around SI genes suggests that efforts to obtain random matings within the crossing block may need to focus on SI diversity and/or overcoming SI (Do Canto et al. 2016). Within IWG, overcoming SI may be easier than other species as it is often self-fertile (Dewey 1978; Jensen et al. 1990), suggesting most desired crosses could be obtained by direct crossing.

## Conclusions

This study used SNP markers generated from low coverage GBS data to assign paternity. Our results show that even with considerable missing data, markers can be used to infer paternity with greater than 74% agreement to the recorded pedigree. While higher assignment rates may be desirable, the observed rate is still sufficiently high to maintain pedigrees, reduce time and labor cost of direct crossing, and increase observed genetic diversity, which should lead to increased genetic gains. For breeding programs of outcrossing species that are utilizing GS, the data required to perform paternity analysis are already generated; thus, paternity analysis can be readily employed. Aside from driving breeding decisions, paternity analysis can also be used to gain insight into the breeding program. While our populations were designed to be a random sample of parental combinations, there was clear evidence that certain cross-combinations were more frequently obtained than others. The results of several GWAS analyses showed evidence that there are genetic factors, including at least one SI gene, that prevent crossing between individual plants which may affect the potential genetic gains of the IWG breeding program. As Li and Brummer (2012) suggest, paternity analysis may be one of the most cost-effective methods to increase breeding efficiency, and with low cost, affordable markers, it is likely paternity analysis will become a standard part of breeding pipelines.

## Electronic supplementary material

Below is the link to the electronic supplementary material.

Fig. S1 Density of single-nucleotide polymorphisms across the 21 intermediate wheatgrass chromosomes (JPEG 794 kb)

Fig. S2 QQ plots for genome-wide association analysis (GWAS) of number of observed progeny in intermediate wheatgrass. Panels represent: **A** GWAS using in silico progeny genotype encoding for a single-loci, self-incompatibility gametophytic system for observed progeny combinations, *n* = 7921. **B** Principal component analysis (PCA) of progeny matrix principal component (PC) 1. **C** PCA of PC 2. **D** PCA of PC 3, *n* = 89 (TIFF 22503 kb)

Fig. S3 Relationship between the total number of inflorescences per successful pollen parent (*x*-axis, *n* = 82) and the number of log offspring (*y*-axis left) and total offspring (*y*-axis right) of intermediate wheatgrass in a polycross breeding program (TIFF 18987 kb)

Fig. S4 Relationship between marker distance in base pairs and linkage disequilibrium *r*^*2*^ in The Land Institute cycle-6 and 7 breeding population (TIFF 16878 kb)

## Data Availability

Original raw sequence data can be found in NCBI sequence read archive (SRA) (https://www.ncbi.nlm.nih.gov/bioproject/) as BioProject accession number PRJNA563706.

## Abbreviations

DUF: Domain-of-unknown function
GWAS: Genome-wide association study
GBS: Genotyping-by-sequencing
GS: Genomic selection
*H*: Shannon’s diversity index
IWG: Intermediate wheatgrass
MAF: Minor allele frequency
NGS: Next-generation sequencing
PAR: Photosynthetically active radiation
PCs: Principal components
PCA: Principal component analysis
SI: Self-incompatibility
SNP: Single-nucleotide polymorphism
SSR: Simple sequence repeat
USP: Ubiquitin-specific protease
