# Supplementary figures and images for: Sequenced-based paternity analysis to improve breeding and identify self-incompatibility loci in intermediate wheatgrass (Thinopyrum intermedium)
Source: Theor Appl Genet. 2020 Aug 12;133(11):3217–33. doi: 10.1007/s00122-020-03666-1 (PMC7547974; doi:10.1007/s00122-020-03666-1)

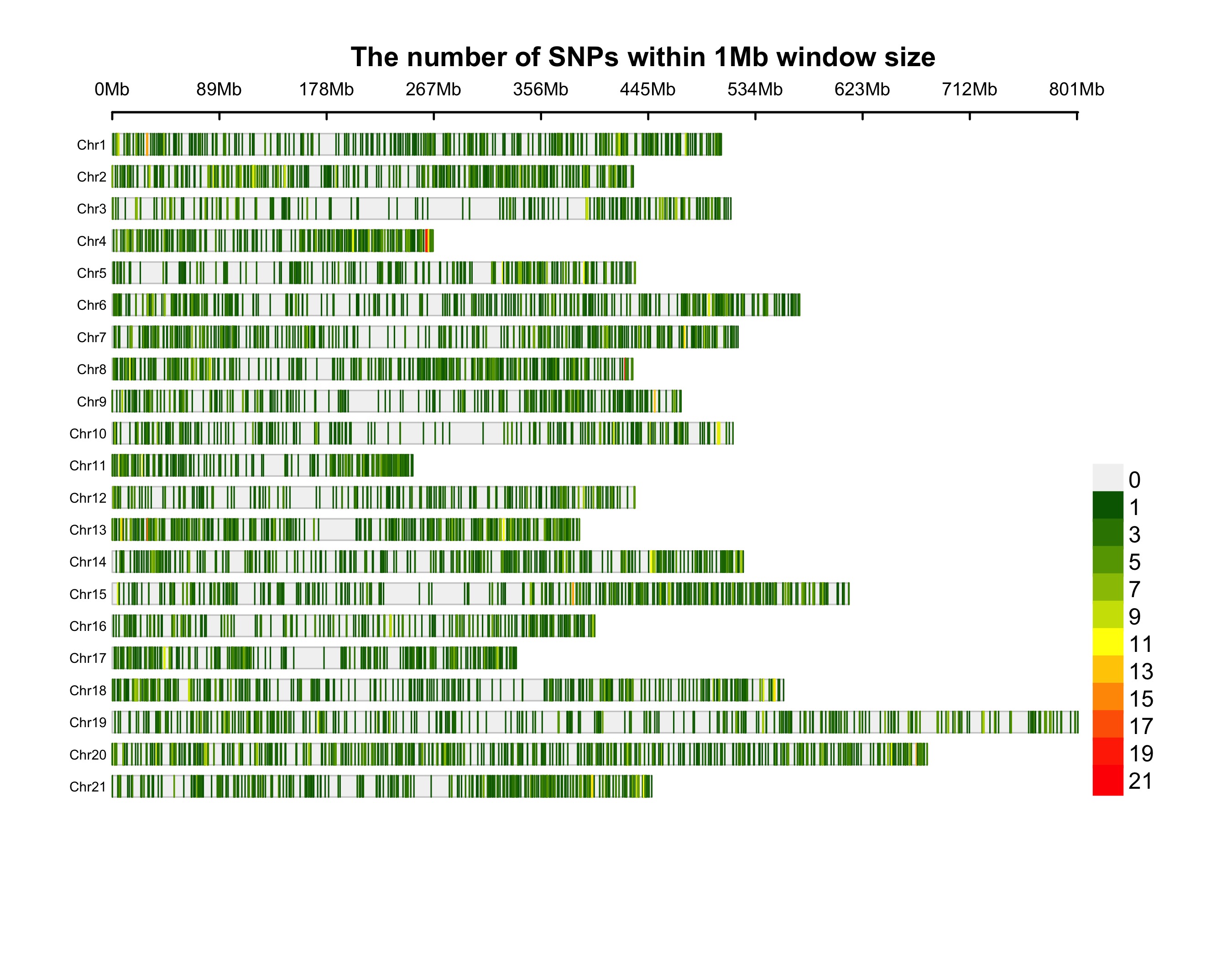

Supplement: Supplementary file 1 — Density of single-nucleotide polymorphisms across the 21 intermediate wheatgrass chromosomes (JPEG 794 kb) [file 122_2020_3666_MOESM1_ESM.jpg]

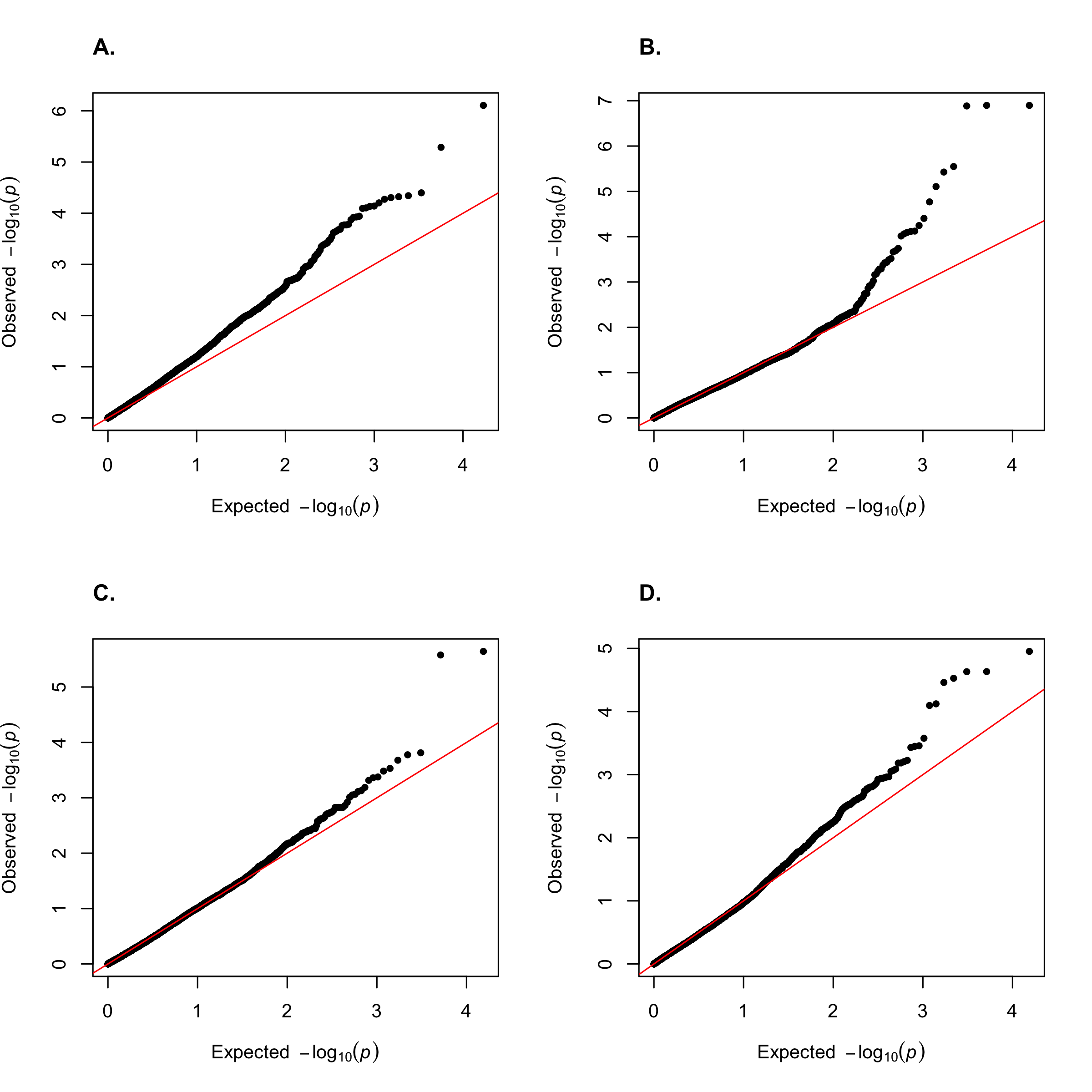

Supplement: Supplementary file 2 — QQ plots for genome-wide association analysis (GWAS) of number of observed progeny in intermediate wheatgrass. Panels represent: A GWAS using in silico progeny genotype encoding for a single-loci, self-incompatibility gametophytic system for observed progeny combinations, n = 7921. B Principal component analysis (PCA) of progeny matrix principal component (PC) 1. C PCA of PC 2. D PCA of PC 3, n = 89 (TIFF 22503 kb) [file 122_2020_3666_MOESM2_ESM.tiff]

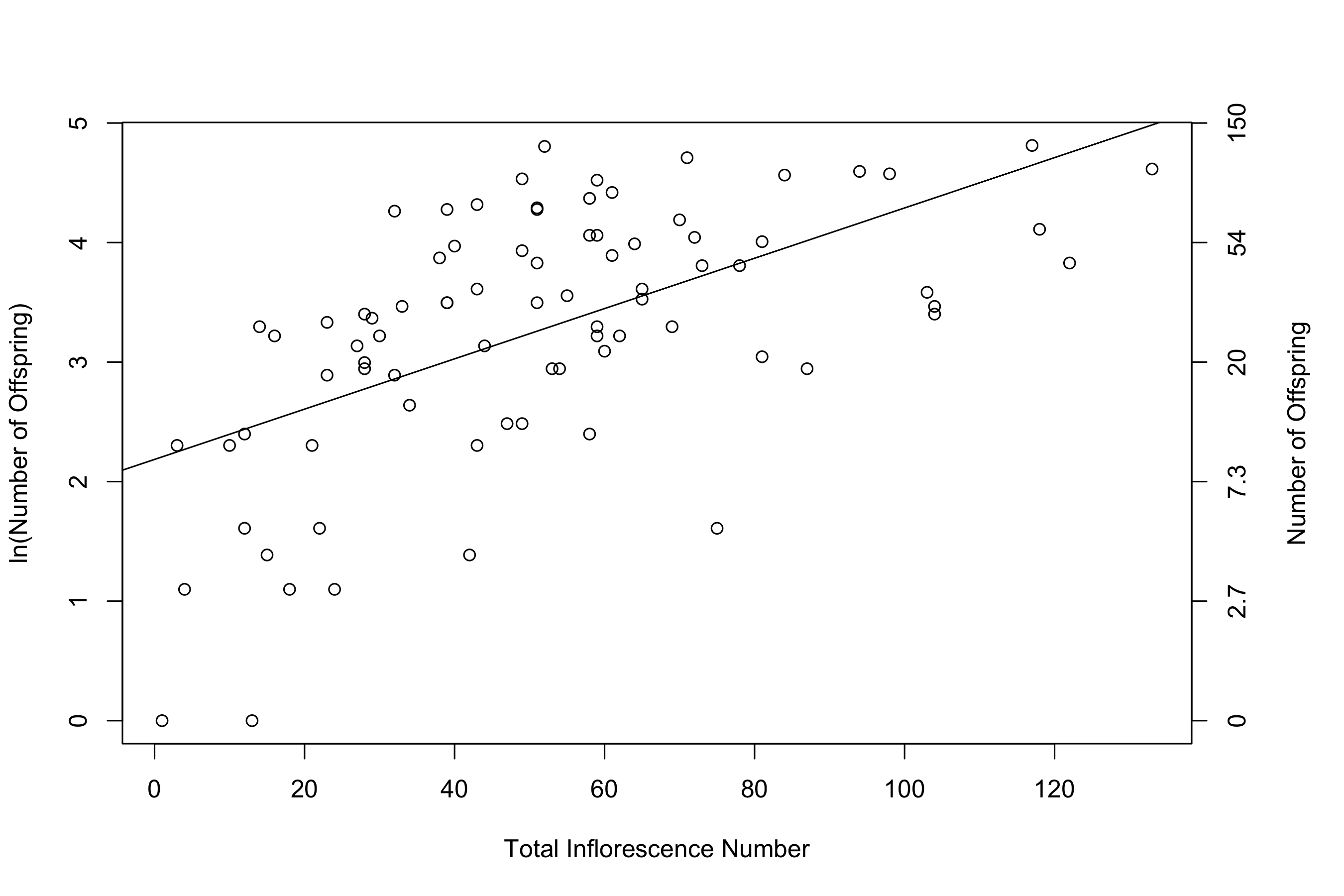

Supplement: Supplementary file 3 — Relationship between the total number of inflorescences per successful pollen parent (x-axis, n = 82) and the number of log offspring (y-axis left) and total offspring (y-axis right) of intermediate wheatgrass in a polycross breeding program (TIFF 18987 kb) [file 122_2020_3666_MOESM3_ESM.tiff]

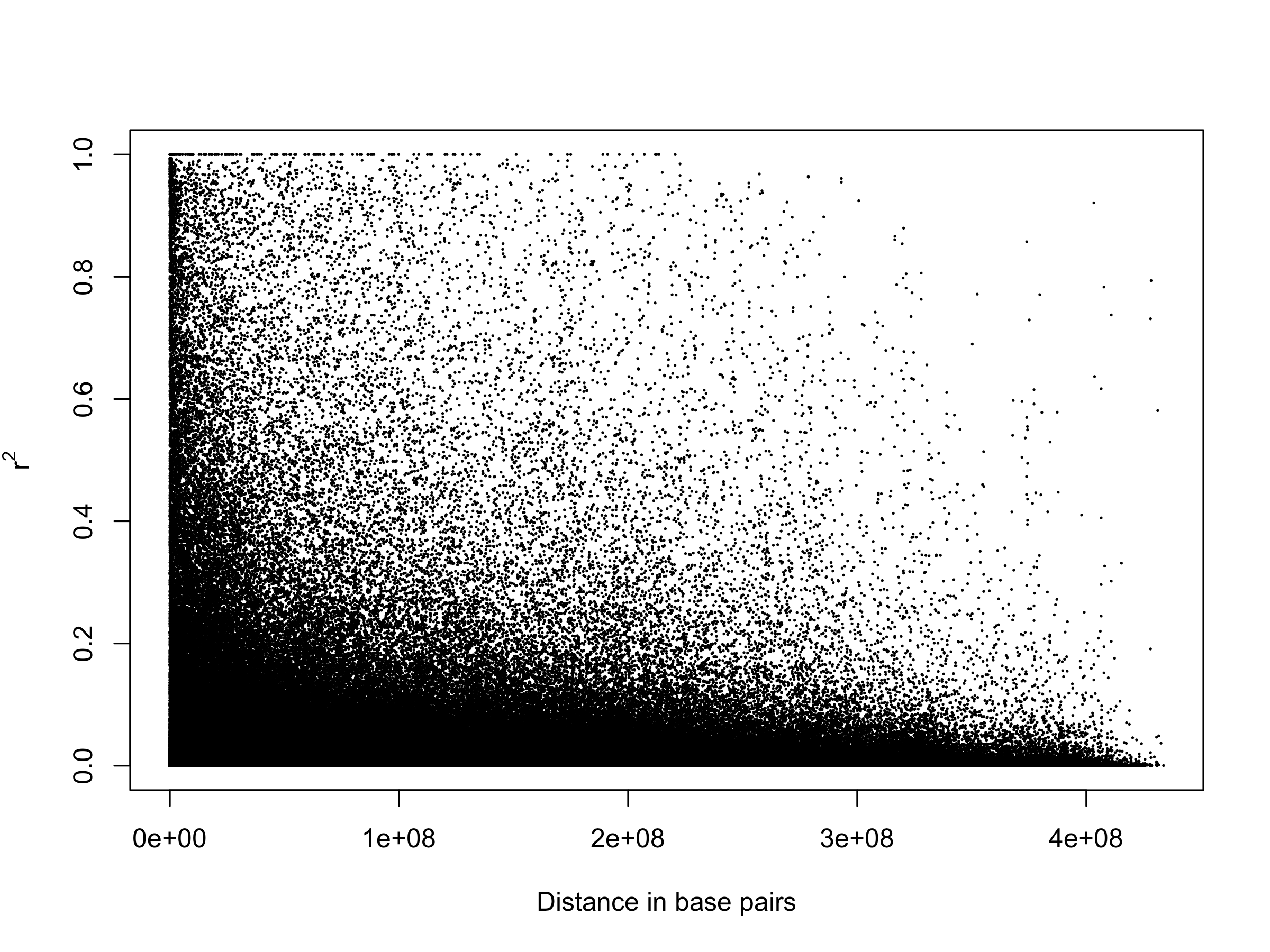

Supplement: Supplementary file 4 — Relationship between marker distance in base pairs and linkage disequilibrium r2 in The Land Institute cycle-6 and 7 breeding population (TIFF 16878 kb) [file 122_2020_3666_MOESM4_ESM.tiff]
